# To Be There or Not to Be There, That Is the Question—On the Problem of Delayed Sampling of Entomological Evidence

**DOI:** 10.3390/insects12020148

**Published:** 2021-02-09

**Authors:** Lena Lutz, Marcel A. Verhoff, Jens Amendt

**Affiliations:** Institute of Legal Medicine, University Hospital Frankfurt, Goethe-University, Kennedyallee 104, D-60596 Frankfurt am Main, Germany; verhoff@med.uni-frankfurt.de (M.A.V.); amendt@em.uni-frankfurt.de (J.A.)

**Keywords:** crime scene, autopsy, cooling period, entomological evidence, expertise

## Abstract

**Simple Summary:**

Proper evidence sampling is at the heart of a sound forensic opinion and failure to follow the standards and guidelines can have serious consequences for the report and expert testimony in court. In casework, forensic entomologists often must base their expert opinion on information about the case and insect evidence provided by third parties, and this presents pitfalls. We analyzed two of those: delayed evidence sampling and the effect of low-temperature storage of the body prior to the autopsy. Our study shows that sampling at the scene is advisable to facilitate a sound entomological report and that the cooling sequence of a corpse must be completely tracked between its removal from the scene until the insect sampling.

**Abstract:**

The aim of the current study was to analyze two major pitfalls in forensic entomological casework: delayed evidence sampling and the effect of low-temperature storage of the body. For this purpose, temperature profiles of heavily infested corpses during cooling and cases in which insect evidence was collected both at the scene and during autopsy were evaluated with regard to species composition and development stages found. The results show that the temperature in the body bags remained at higher average temperatures up to 10 °C relative to the mortuary cooler, therefore, sufficient for larval development, with significant differences in temperature between larval aggregations on one and the same body. In addition, we found large differences both in species number, species composition, and the developmental stages found at the scene and during the autopsy. These data and observations underscore the importance of sampling evidence at the scene and recording temperatures throughout the cooling period of a body.

## 1. Introduction

Forensic entomology i.e., the use of insect evidence in legal investigations [1], has become one of the most accurate and precise methods to establish the minimum post-mortem interval (PMI_min_), i.e., the time since the first insect colonization on a body, in the later stages of decomposition [2,3,4,5,6]. In addition to the worldwide scientific development in this field, with an average of approximate 100 publications per year since 2013 (Web of Science, 12 August 2020), forensic entomology is now more recognized in forensic casework by law enforcement: entomological reports are now an integral part of court proceedings in Europe [7,8,9,10,11], North [12] and South America [13], Asia [14,15,16,17], Africa [18], Australia and New Zealand [19], and the Middle East [20]. To present high quality entomological findings in the court, various standards and guidelines for sampling, analyzing, and reporting entomological evidence have been published in recent years [21,22,23]. These “rules” are usually easy to follow when a forensic entomologist is involved in a crime investigation from the beginning, but the reality in casework is different and it is sometimes far from “best practice” [24,25,26]. A forensic entomologist often must write a report based on the information provided to them regarding the case (photographs, videos, police reports) rather than first-hand experience, and after examination of insect evidence sampled by third parties, such as death investigators, medical examiners, or non-medical professionals. This could lead to a number of problems in the collection of insect evidence, e.g., missing the oldest developmental stage [24], contamination [27], or incorrect sample handling [28,29]. One of the biggest problems is that evidence is frequently not sampled at the scene but during the autopsy [24,28,30,31,32,33,34], often several days after the discovery of the body [31,32,35,36]. An analysis of 127 cases from the Harris County Institute of Forensic Sciences found that autopsy only sampling was performed in 42% of the cases while sampling at the scene and during the autopsy occurred only in 2% [24]. To our knowledge, no publications analyze how much the evidence collection differs between the scene and the autopsy on one and the same case. Sampling only at the autopsy may lead to a delay of several days before evidence collection, which could affect not only the composition of the entomological findings, but their stage of development. The impetus for this study was a 2018 death investigation that highlighted the importance to PMI_min_ estimates of who takes entomological samples and when. 

### Case Study

On 14 November 2018, the body of a 49-year-old man was found in an apartment after the janitor of the building complex reported a bad smell from his apartment. The body was in an advanced stage of decomposition with larval infestation, in supine position on the floor wearing just a T-shirt. In the apartment, paraphernalia (e.g., syringe and spoon) for drug use were found. The flat was in a poor state of cleanliness, the window was in a tilted, slightly open position and the heating was on. In addition to a medical examiner, a forensic entomologist was at the scene for sampling insect evidence. On the body itself, third instar larvae of blow flies (Diptera: Calliphoridae) were found and fresh, light colored pupae underneath a carpet close to the body. The samples were transferred on the same day to the insect laboratory. Half of the larval sample was killed with almost boiling water and then stored at 96% ethanol for length measurement and species identification, while the remaining larvae plus the sampled pupae were incubated at 25 °C until the emergence of the adult flies. Meanwhile, the body was stored in a plastic body bag in a cooler at 4 ± 2 °C.

On 19 November, i.e., five days after discovery of the body, an autopsy was performed and insect evidence (third instar larvae of blow flies), was collected. The insect samples from the autopsy were handled in the same way as those from the scene. The cause of death could not be determined in the autopsy due to the advanced decomposition of the body, but toxicological analyses showed an intoxication. The entomofauna of the body included larvae and pupae of *Calliphora vicina* and larvae of *Calliphora vomitoria*, *Lucilia ampullacea,* and *L. sericata. Calliphora vicina* was the numerically dominant species, accounting for 90% of the specimens. The species composition was almost identical at the scene and autopsy, except for piophilid larvae which were found on the body only during the autopsy. However, the crucial difference between the insect evidence from the scene and autopsy in relation to establishing a PMI_min_ was provided by the pupae from the scene. Due to the lack of sound temperature measurements at the scene, two different temperatures (20 °C and 25 °C) were used as the basis for the development of the most important species here, *C. vicina*, which were intended to reflect a range of possible room temperatures in the apartment. The PMI_min_ for the scene data was estimated to be 8–10 days, while using the data from the autopsy resulted in a PMI_min_ of 3–4 days. In addition to this large difference in the estimated PMI_min_ due to the different developmental stages, the sampled larvae also differed significantly in length (df = 64.98, *p* < 0.001), with the larvae from the scene being on average 3.3 mm longer than the larvae from the autopsy. 

In addition to the incomplete and fragmentary insect evidence when obtained only during the autopsy, a second problem arises, namely the cooling time before the autopsy [34,37]. As already mentioned by Charabidze and Hedouin [36], temperature is still a weak point in forensic entomology due to numerous factors that influence the temperature the larvae are exposed to during their growth. Many studies have tried to establish the most accurate guidelines possible for temperature reconstruction and estimation [36,38,39,40,41,42,43,44,45], but all of them concentrate only on the temperature history prior to the discovery of the body. The time between the removal from the death scene and the autopsy is still a “gap of knowledge” when it comes to accurate temperature estimation and consideration of this cooling period on the development of forensically important species. Although there have been many studies on the influence of refrigeration on the development of necrophagous insects [28,37,46,47,48,49], data on the temperatures of heavily infested bodies inside body bags during the storage prior to an autopsy are still scarce, with only one study published [34]. 

The current study highlights two of the biggest problems in forensic entomological casework, i.e., evidence sampling and body storage temperature. Firstly, we analyzed the temperature profiles of heavily infested bodies stored in body bags in a walk-in cooler to describe the effect of cooling on the temperature inside the bags, i.e., on the temperature to which the larvae were exposed. Secondly, we analyzed the effect of who collects the insect evidence and when, on evidence composition (species and developmental stages), by examining cases in which insect evidence was collected both at the scene and the autopsy. Overall, common pitfalls are presented with data from everyday casework, which nourish and support the need for laboratory studies, along with guidelines for dealing with these pitfalls. 

## 2. Materials and Methods

### 2.1. Body Cooling

We evaluated the temperature profiles of eight bodies during the summer months from May until August 2017. After the discovery of the bodies and a first examination at the scene, they were placed in white plastic body bags and transported to the Institute of Legal Medicine Frankfurt. All bodies were heavily infested with insects and stored in a walk-in cooler after their arrival at the institute. The walk-in cooler was set to a baseline temperature of 6 ± 2 °C. The day and time of delivery of the body to the institute and the collection of the body by the mortician, i.e., the beginning and end of cooling, were noted. As soon as possible after arrival, prior to the temperature measurements, the bodies were inspected, i.e., pictures were taken, the number of maggot masses was noted, and the insect evidence was sampled. Since the examination of the bodies had to be included in the routine activities of the institute, the evaluations and measurements for research purposes could not always be carried out immediately after the arrival of the bodies, but only on the next day or the Monday after the weekend. For this reason, the actual temperature measurement on the bodies may differ from the entire cooling period of the body (Table 1). For bodies that were cooled more than 6 days (n = 2), insect samples were also taken a second time during cooling to examine the effect of cold storage on larval development. 

The temperature was measured hourly with three iButtons (DS1922L-F5, Maxim Integrated, San Jose, CA, USA) placed in the body bags. If visually clearly defined maggot masses were present, the iButtons were placed directly into them, otherwise they were located at sites of moderate maggot aggregations. For this reason, the position of the iButtons differs between the examined bodies (Table 1). Before placing the data logger in the body bags, the initial temperature of the walk-in cooler close to the body was noted/confirmed. The temperature profiles of all eight bodies were visually analyzed and the temperature difference of the three body parts for each of the cases was tested for significance using a Kruskal–Wallis test (*p* = 0.05). The insect samples, i.e., third instar blow fly larvae, were killed with almost boiling water and then stored in 96% ethanol. Species identification was performed based on morphological characters with the current systematic literature [50]. In cases where samples of two events were available, i.e., after a short (14 and 40 h) and a long (160 and 280 h) cooling period, the length of 100 maggots of each event was determined using a geometric micrometer [51].

### 2.2. Scene vs. Autopsy

We evaluated insect-associated cases from the Institute of Legal Medicine Frankfurt in which entomological evidence was sampled at the scene and during the autopsy from November 2018 until October 2020 (n = 29). In most cases, the sampling at the scene was performed by a medical examiner as part of the postmortem inspection, and sampling during autopsy was performed by a forensic entomologist. Part of the fly larvae were killed with almost boiling water and afterwards stored in 96% ethanol, and the remaining specimens were transferred to minced meat and bred under controlled temperature in the laboratory until the adult stage. Adult specimens as well as beetle larvae were killed by freezing at −20 °C and then stored in 96% ethanol. Species identification was performed on the basis of morphological characters with the current systematic literature [50,52] and voucher specimens. Besides species identification, information on the developmental stages found at the scene and the autopsy was noted. For cases (n = 13) where a sufficient number of maggots was present, for both samples, scene and the autopsy, the larval length was measured with a geometric micrometer [51]. In addition, the date (day, month, year) of discovery and autopsy, the sex and age of the deceased, the place of discovery (indoor or outdoor), the presumed PMI, and the type of death (natural, unnatural, unclear) were noted for each case. We used a paired *t* test to examine the difference in larval length for samples from the scene and the autopsy. All data were analyzed and charted with R version 3.6.2 [53].

## 3. Results

### 3.1. Body Cooling—Temperature Profiles

**Figure 1:**
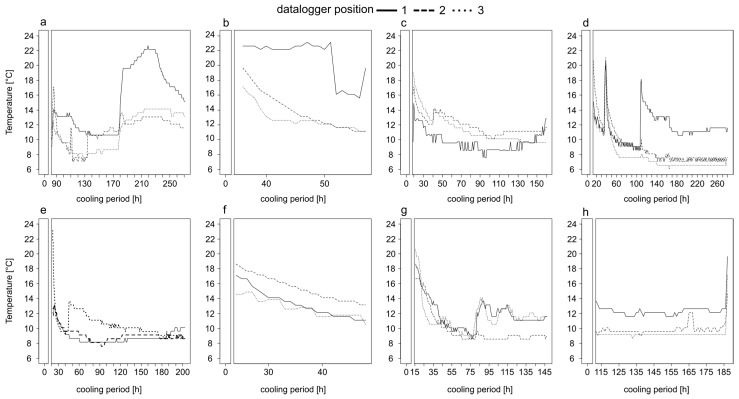
Temperature profiles of bodies heavily infested with insect larvae stored in a walk-in cooler in the Institute of Legal Medicine Frankfurt from May until August 2017. Information on the exact position of the iButton on the body, the period of temperature measurement and the entire cooling period are given in Table 1. The temperature profiles belong to corpse 1 (**a**), corpse 2 (**b**), corpse 3 (**c**), corpse 4 (**d**), corpse 5 (**e**), corpse 6 (**f**), corpse 7 (**g**) and corpse 8 (**h**).

All eight temperature profiles inside the body bags showed higher average temperatures (Table 2, Figure 1) of up to ≈10 °C more, than the baseline temperature of 6 °C of the walk-in cooler, independent of the duration of temperature measurement, the entire cooling period, or the position of the iButton on the body. 

In most cases, there was a steep decline in temperature for the first 50 h and then the temperature fluctuated (±2 °C) around a minimum (Figure 1). The temporal decrease to the minimum temperature inside the body bags took, depending on the case, from ≈80 h (Figure 1e,g), over ≈100 h (Figure 1c) up to ≈140 h (Figure 1d). However, in most cases the temperature did not reach a stable minimum, even after long cooling periods of 271 (Figure 1a) or 280 h (Figure 1d). Overall, the temperature profiles were highly variable over time (Figure 1). In the two cases of short cooling periods of up to 57 h the temperature decreased on average with a rate of 0.25 °C per hour (Figure 1b,f). 

### 3.2. Body Cooling—Temperature Difference of Body Positions

**Figure 2:**
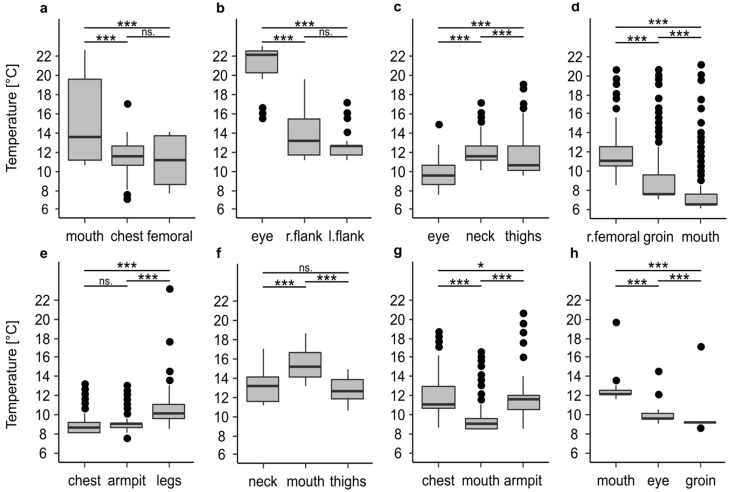
Temperature differences between the maggot masses on bodies stored in a walk-in cooler. Information on the period of temperature measurement and the entire cooling period are given in Table 1. The temperature profiles belong to corpse 1 (**a**), corpse 2 (**b**), corpse 3 (**c**), corpse 4 (**d**), corpse 5 (**e**), corpse 6 (**f**), corpse 7 (**g**), and corpse 8 (**h**). The asterisks representing *p*-values with *** <0.001, ** <0.01, * <0.05, *n.s* not significant.

In addition to the high variability of the temperature profiles within every single profile, there were large differences between the temperature profiles of the recorded positions on one and the same body (Figure 1 and Figure 2, Table 2). In all cases, at least two of the three recorded positions were significantly different to each other (*p* < 0.001), which in extreme cases resulted in the mean temperature at one recorded position being 8.1 °C or 7 °C warmer than at the other positions. In 50% of all cases (Figure 2c–f) the temperature differed significantly (*p* < 0.001) between all positions. There was no clear trend that a certain position of a body, i.e., eye or oral cavity, always has the highest temperature. 

### 3.3. Body Cooling—Temperature Difference of a Body with and without Larvae

**Figure 3:**
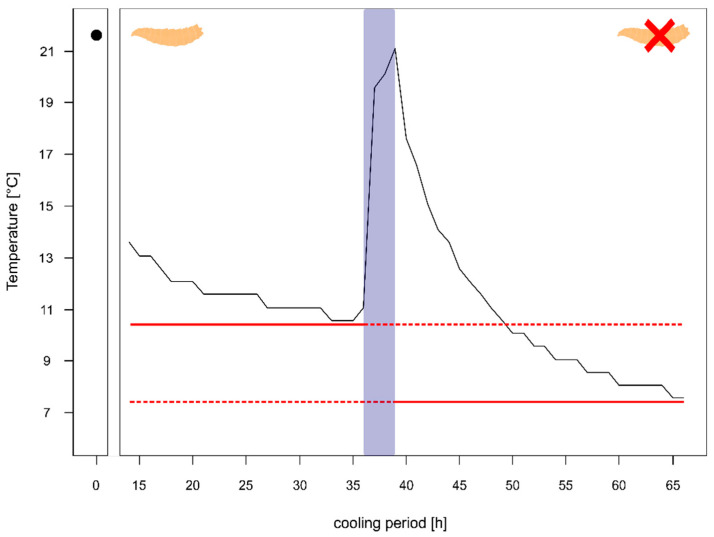
Temperature profile of a body prior to autopsy (with heavy larval infestation and several larval aggregations) and after the autopsy (almost no larvae left). The red solid line shows minimum temperature of the body prior autopsy and after the autopsy, respectively. The blue box represents the temperature measurements during the autopsy. The maggot represents the body with heavy larval infestation and the maggot with the red X represents the body after the removal of larvae.

For one of the corpses (corpse 4) we had information on the temperature in the body bag prior to the autopsy and even after the autopsy. The comparison of the temperature after 24 h of cooling with maggot masses on the body (left side Figure 3) showed that the decrease over time happens quite slowly with a rate of 0.12 °C per hour and results in a high minimum temperature of 11.07 °C, which is ≈5 °C above the cooler temperature.

Whereas after the autopsy, when the body was washed and most larvae were removed, the decline in temperature was faster with 0.41 °C per hour resulting in a minimum temperature of 7.56 °C after 24 h (right side Figure 3), being just 1 °C warmer than the overall minimum temperature during the entire cooling.

### 3.4. Body Cooling—Entomological Evidence and Larval Length

Overall, seven blow flies: *C. vicina*, *C. vomitoria*, *Ch. albiceps*, *L. ampullacea*, *L. sericata*, *P. regina*, *P. terraenovae*, and one flesh fly namely *S. argyrostoma* were identified (Table S1). The most common species was *L. sericata*, being present on each of the eight bodies. All measured temperatures (Table 2) were sufficient for larval development of *C. vicina*, *C. vomitoria*, *P. terraenovae* and *L. sericata* inside the body bags, assuming a lower threshold of 2 °C, 3 °C, 7.8 °C and 9 °C respectively [54].

In two cases, we were able to compare the larval lengths after both a short and a long cooling period. All larvae belonged to *L. sericata*. The comparison showed that in one case the specimens underwent significant growth during the cooling (Figure S1a). The larvae with a short cooling period of 40 h were on average 2.52 mm shorter than after 160 h of cooling (df = 191.77, *p* < 0.005). In the second case we found a significant (*p* < 0.001) decrease in larval length (Figure S1b). The larvae were on average 1.46 mm shorter after 280 h of cooling than after 14 h.

### 3.5. Scene vs. Autopsy

In total, we investigated 29 cases from November 2018 to October 2020. Of the 29 cases, 72.4% (n = 21) were male and 27.6% (n = 8) female with an average age of 66.4 years. All bodies were found indoor and the majority (75.9 %, n = 22) were found during the summer months from April until September whereas 24.1% (n = 7) were found from October until February. The suspected PMI averaged 17 days, with two cases showing a rather long PMI ranging from three months (case 26) to two years (case 13). An autopsy was performed on average 6.2 days after the bodies were found. In more than 50% (n = 17) of the cases the autopsy could not find a clear cause of death due to the advanced decomposition of the body. In 37.9% (n = 11) of the cases the person died of natural causes (e.g., heart failure, internal causes) and in one case the person died of unnatural causes by intoxication.

### 3.6. Scene vs. Autopsy—Entomological Evidence

Overall, we found a large difference both in species number, species composition, and the developmental stages found at the scene and during the autopsy (Table 3). Only in 6.9% (n = 2; case 8, 14) of all cases exactly the same species were found. In 93.1%, the species composition was very different from each other, in an extreme case (case 15) even with a difference of up to six species between the entomological evidence taken at the scene and during autopsy. There was a general trend that more species were found during the autopsy. The development stages found, were identical between the scene and autopsy in more than 50% of the cases, but in almost one third of these cases the PMI was around 7–10 days, so that probably only one developmental stage (larvae) was available. In 48.3% of the cases, we saw differences in the developmental stages found, with a slight trend that older developmental stages such as pupae or puparia were found at the scene but not during the autopsy. Considering both the species found and the stages of development together, there were differences in 96.5% (n = 28) of all cases resulting in just one case (case 14) where the entomological evidence (insect species, developmental stage) was exactly the same both at the scene and at the autopsy.

### 3.7. Scene vs. Autopsy—Larval Length

For 13 cases we had a sufficient number of larvae both from the scene and the autopsy to compare the larval length. In eight cases larvae of *L. sericata* were measured, in four cases, larvae of *C. vicina*, and in one case, larvae of *Ch. albiceps.*

**Figure 4:**
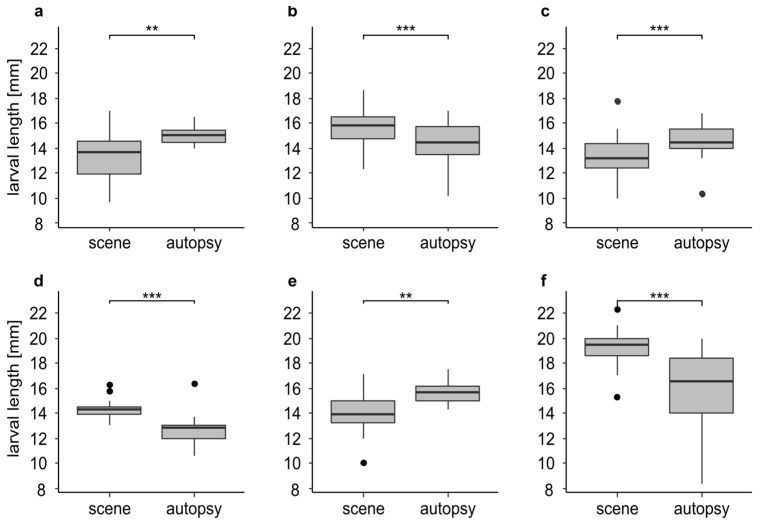
Larval length in mm for insect samples from the scene and the autopsy (n-values = identical for the scene and the autopsy; (**a**) larvae of *L. sericata* (n = 20) from case 3 after 0 and 8 days of cooling; (**b**) larvae of *L. sericata* (n = 60) from case 5 after 0 and 8 days of cooling; (**c**) larvae of *L. sericata* (n = 44) from case 23 after 0 and 5 days of cooling; (**d**) larvae of *L. sericata* (n = 13) from case 24 after 0 and 4 days of cooling; (**e**) larvae of *L. sericata* (n = 14) from case 27 after 0 and 8 days of cooling; (**f**) larvae of *C. vicina* (n = 50) from case 1 after 0 and 5 days of cooling. The asterisks representing *p*-values with *** <0.001, ** <0.01.

We found significant differences in larval length in 46.1 % (n = 6) of the cases (Figure 4). In three cases (Figure 4a,c,e) the larvae underwent significant development during the time between the discovery of the body and its autopsy. In these cases, the larvae from the autopsy were significantly larger (*p* < 0.01) with a mean difference of up to 1.8 mm (Figure 4e). In the other three cases (Figure 4b,d,f) we found a significant (*p* < 0.001) decrease in larval length. The larvae from the autopsy were significantly smaller with a mean difference of up to 3.2 mm (Figure 4f). In 53.9 % (n = 7) of the cases we found no significant differences in larval length (Figure S2).

## 4. Discussion

This study presents real case data on two major pitfalls in forensic entomological casework: firstly, the thermal history of maggots on a body, i.e., the effects of storage in a cooler prior to the autopsy, and secondly the sampling of insect evidence with regard to the place and time of the sampling (at the scene right after the discovery of the body, or during the autopsy), as well as the training of the person performing the sampling. 

### 4.1. Body Cooling

Knowing and understanding temperature is a key element in forensic entomological casework due to its effect on carcass decomposition, activity, oviposition, and succession of insects on a cadaver and especially on the growth of forensically important species. Therefore, the documentation and estimation of temperature insects experience during their growth is important, recommended in every standard book of forensic entomology, and crucial for an accurate PMI_min_ estimation. One temperature-relevant aspect that has received little attention so far is the storage of the insect-infested body in a cooled morgue prior to the autopsy, which is of major importance especially in cases where the entomological evidence is obtained just during the autopsy. Even in quite recent guidelines for the use of temperature in casework [36], the use of a margin error of ±2 °C on the refrigerator temperature is given as a solution. Our results showed that all temperature profiles inside the body bags showed average temperatures that were up to 10 °C higher than the baseline temperature of the walk-in cooler, no matter the duration of the temperature measurement, the entire cooling period or the position on the body. Even the lowest mean average temperature was 1.8 °C higher than the cooler. Therefore, adding just 2 °C to the temperature of the cooler will possibly not reflect the actual temperature the larvae experienced during growth and could lead to an erroneous PMI estimation depending on the duration of the impact. Our results are in line with Huntington et al. [34] and Thevan et al. [55] who reported a significant temperature difference of up to 8 °C between the inside of the body bag and the storage cooler’s temperature. 

These major temperature differences are caused by the maggot masses on the body, which have been reported to exceed the surrounding environment in the field much as 10–30 °C [56,57] and maintain temperature even under cooling [58]. This effect can be clearly seen when we compare the temperatures of one and the same body with and without maggots. The decline in temperature without maggots was much faster (0.41 °C/h) and reached a stable minimum close to the cooler temperature after 24 h, while the decline in temperature with maggots was very slow (0.12 °C/h) and reached a minimum temperature still 5 °C higher than that of the cooler. In our study, we focused not only on temperature profiles in the body bags, but also on recording the temperature inside the maggot masses during cooling, and especially on the variability on one and the same body. Our results show that in all cases, at least two of the three recorded maggot masses were significantly different to each other which, in extreme cases, resulted in the mean temperature at one recorded position being 8.1 °C or 7 °C warmer than at the other positions.

This problem is further complicated by the fact that specimens of different ages and sizes can withstand cooling quite differently [48,59], and flies may be able to handle the temperature decrease at least temporarily with their behavior or physiology [60]. Diapause, marked by the reduction of metabolic activity, is mainly regulated by the photoperiod acting on the maternal generation of blow flies and by the thermal conditions of development of their larvae. Vinogradova and Reznik [61] showed the occurrence of diapause in *C. vicina* field populations already from the middle of August, with temperatures relevant for the larvae in the low double-digit range. Hence, depending on the time of the year (day length) and the duration of the cold storage (temperature) larval diapause cannot be ruled out during storage in the morgue. This might be no serious problem in summer and especially during further breeding of living specimens after sampling at a temperature of normally >20 °C, as specimens might acclimatize quickly and terminate diapause. However, we admit that knowledge about species-specific induction and termination of diapause (and dormancy) in blow flies is still limited. Due to these kinds of impacts and variations and the fact that the actual dynamics are more complex [62], it will be difficult or even impossible to establish a serious correction factor for calculating the temperature experienced by the larvae during storage, not least as not all of them have experienced the same temperature [34]. Nevertheless, it is important to know about the temporal decline of temperature inside a body bag after the transfer to the cooler, i.e., the period larvae experience still high temperatures. The cooling rate of a body without insect infestation is, depending on the weight, 1–2 °C per hour [63]. Our results showed that the cooling rate of a heavily infested body is around 0.12 °C per hour and that it can take up to 100 h until the temperatures inside the bag reaches its minimum - even the lowest temperature inside the body bag is usually higher than the cooler temperature. Does that special kind of temperature regime have an effect on maggot development? Our results show that blow fly larvae can undergo significant development despite the cooling, resulting in a difference in length of up to 2 mm, which can lead to erroneous PMI estimations depending on species and temperature. These results are in line with other studies [34,55], according to which maggots clearly continue to develop during cooling and even complete their development. In our study, comparison of scene- and autopsy-based PMI_min_ estimations based only on larval length (results not shown) indicates that discrepancies are less than 24 h even for significantly different larval lengths and therefore likely to be negligible, as PMI_min_ is usually narrowed down to the day. However, further studies need to be conducted to quantify the effect of cooling on different species and their developmental stages.

The ongoing developmental and feeding activity of the insects along with the decay of the body is not just a problem for the forensic entomologist, but also for the medical examiner. Since a high larval activity and progressing decay can impact autopsy findings [64] due to substantial tissue loss [34]. Especially maggot masses around the head i.e., in the mouth, the eyes, and around the neck, can easily destroy signs of bruises or skin lesions that would be helpful to determine a cause of death. We have seen some cases where the corpses at the scene were just in an early stage of decomposition, with beginning larval aggregation on the head, but at the autopsy several days later all the tissue of the face was lost, and the head was almost skeletonized. We believe that these problems are more common than expected, especially in the summer and recommend that heavily infested bodies be autopsied as soon as possible.

Besides further growth, i.e., an incline in larval length during the cooling, we also saw the opposite i.e., a decline in larval length of up to 3.2 mm. This can probably be explained by the developmental stage that the specimens on the body had already reached before storage [48]. Archer et al. [28] found that late third instar larvae of *C. vicina* shrink on average of −1.2 mm during cooling, whereas second and mid third instar larvae grow overall during cooling. If the maggots on the body are already in post feeding stage then they could be preparing for pupation during cooling, which in general is associated with larval shrinkage of several mm depending on species and temperature [65]. An example of this phenomenon is provided by the case study presented in the introduction. The majority of larvae at the scene was probably just before post-feeding, resulting in the maximum length during their development. Normally, there is a decrease in larval length during post feeding until the onset of pupation. During the cold storage until the autopsy, the larvae of the cold adapted species *C. vicina* continued its development. Reiter [66] observed shrinkage of 2.9 mm at 6.5 for *C. vicina* mm during this phase in the development, which is in a similar range to our results. For casework, it is therefore necessary to know at least the prevailing development stages prior to cooling and a subsequent preservation. 

### 4.2. Scene vs. Autopsy

Proper evidence sampling is at the heart of a sound forensic entomologist’s opinion and failure to follow the protocol can have serious consequences for the report and expert testimony in court [28]. Hence, the gold standard is the sampling of entomological evidence at the scene and during the autopsy [21], and in cases of low population due to season or accessibility of the body, even only scene collection by a trained expert may be sufficient—but this will be the exception rather than the rule: many studies demonstrated that insect evidence is frequently sampled just during the autopsy [24,28,30,31,32,33,34] and sometimes even several days after the discovery of the body [31,32,35,36]. Our results show that such practices can lead to large differences in species diversity and the developmental stages found, leading to a biased or wrong entomological report. Missing the oldest developmental stage will lead to erroneous and especially compromised interpretation of the entomological evidence. As shown in the case study (Figure S3), the lack of pupae, i.e., the oldest developmental stage, at the autopsy, resulted in an underestimation of the PMI_min_ of up to 5 days, depending on the temperature used for the estimation. This difference will become larger the older, i.e., further developed, the missed developmental stage is. In our study, using entomological evidence collected only at autopsy would have led to an underestimation of the PMI_min_ in 24.4% (n = 7) of cases. 

Additionally, our survey revealed that during the autopsy more species were found. This is probably because in our study a trained forensic entomologist took the samples during the autopsy, while a medical examiner was in charge at the scene. Reasons for poor evidence sampling can be the lack of experience of the persons in charge at the scene of death (police, crime scene technicians, medical examiner), the lack of time and, above all, the unawareness of the diversity of entomological evidence. However, to have just a limited view on the species composition on a body, due to, e.g., missing indicator species for certain stages of decomposition, seasons of the year, or for post-mortem transfer, can have a major effect on PMI estimation and case evaluation. 

## 5. Conclusions

Forensic entomology works with live animals that interact constantly and closely with their environment and faces a diversity of species and morphological manifestations that is difficult for an entomological non-expert to handle. It therefore must deal with many pitfalls when it comes to casework and knowing them will decrease their negative impact.

Our study shows that sampling at the scene is advisable if not mandatory to facilitate a sound entomological report. Most sub-optimal sampling happens because of the lack of knowledge and experience. As a forensic entomologist will not attend every scene, educating and training the police, crime scene technicians and medical examiners is one important approach to cope this issue. 

If on-site sampling has not occurred, the first samples should be taken immediately upon arrival of the body at the mortuary, even (or especially!) if the autopsy is not performed until days later. During autopsy, sampling should focus not only on the body itself but also on the body bag, the clothes and other belongings of the deceased as the fauna perhaps responded to the changed environment and temperature and left the body for shelter or preparing for pupation. 

A better understanding of the influence of (rapidly and drastically) fluctuating temperatures on the growth of necrophagous insects is urgently required in forensic entomology. For the evaluation of the entomological evidence and writing the report, the temperature profile and cooling sequence of the body must be completely tracked from its removal of the scene until the insect sampling. If possible, temperature measurements in the body bags itself but more important in the larval aggregation should be carried out. In the absence of these data, the cooling and resulting slower growth should be discussed in the report, but always the fastest possible development (i.e., without cooling effects, for example) should be calculated to meet the concept of PMI_min_.

Even though this study is not based on a designed experiment, but analyzes data from everyday casework, it is very well suited to illustrate current problems, particularly in temperature reconstruction and evidence collection. It presents detailed data that ultimately demonstrate the need to track the temperature profiles of stored bodies and sample insect evidence both at the scene and during autopsy. Most importantly, it is a clear call and provides a basis for further research focusing on the link between laboratory studies and casework.

Returning to the question of “to be there or not to be there”; it is always better for the forensic entomologist to be at the scene and sample the evidence by him or herself, but this will not be a regular option. We just need to know then what to do if we were not there, and what information is mandatory.

## Figures and Tables

**Table 1:** Information of the bodies (n = 8) used for the temperature profiles in the walk-in cooler. “temperature measurement” refers to the actual duration of the measurement with the temperature logger on the bodies, while “cooling period” describes the time between the arrival of the body at the institute, i.e., beginning of cold storage, and the collection of the body by the mortician, i.e., end of cold storage. N° is the number of body and n the sample size.

N° Body	Maggot Masses [n]	Position of Temperature Measurement on the Body			Temperature Measurement [h]	Cooling Period [h]
		1	2	3		
1	1	mouth	chest	femoral	189	271
2	6	eye cavity	right flank	left flank	22	57
3	1	eye cavity	right neck	thighs	142	160
4	2	femoral	groin	mouth	267	280
5	4	chest	armpit	legs	192	213
6	2	left neck	mouth	thighs	25	48
7	2	chest	mouth	armpit	142	157
8	3	mouth	eye cavity	groin	76	187

**Table 2:** Temperature difference between the three positions used for temperature measurement on each body. Information on the exact position and measurement periods are given in Table 1. “Temperature” shows the mean values over the measurement period with standard deviation; “Initial temperature” is the temperature in the cooler directly before the start of the measurements in the body bags. Its variation is explained by the routine activity and different opening and closing times of the walk-in cooler in the context of the delivery and examination of other bodies.

N° Body	Initial Temperature [°C]	Temperature [°C]		
		iButton 1	iButton 2	iButton 3
1	8.5	15.4 ± 4.2	11.3 ± 1.9	11.1 ± 2.5
2	9.4	20.9 ± 2.8	13.9 ± 2.6	12.8 ± 1.6
3	11.3	9.7 ± 1.3	11.9 ± 1.3	11.4 ± 2
4	9.2	11.6 ± 1.9	9.0 ± 2.6	7.8 ± 2.3
5	7.0	8.9 ± 0.9	9.0 ± 0.9	10.4 ± 1.6
6	15.2	13.3 ± 1.8	15.4 ± 1.7	12.8 ± 1.2
7	-	11.9 ± 1.9	9.9 ± 2.2	11.58 ± 2.0
8	8.3	12.4 ± 0.9	9.9 ± 0.8	9.3 ± 0.9

**Table 3:** Summary of cases where entomological samples were taken at the scene and during autopsy from November 2018 to October 2020. Information on the number of insect species is given. In brackets species unique to the scene or autopsy are listed. The stages of development found, where A= adult specimen, PR = puparia, P = pupae, L = larvae, are listed. In bold are all cases where the larval length was measured. The check mark indicates if a developmental stage was found and the dark gray filling if it was missing.

Scene						Autopsy				
A	PR	P	L	Species [n]	Case	Species [n]	L	P	PR	A
		✓	✓	4 (0)	**1**	5 (1)	✓			
	✓		✓	4 (1)	2	3 (0)	✓	✓	✓	
		✓	✓	2 (1)	**3**	3 (1)	✓	✓	✓	
		✓	✓	5 (0)	4	5 (0)	✓			
	✓	✓	✓	3 (1)	**5**	5 (3)	✓			
	✓		✓	1 (0)	6	2 (1)	✓	✓	✓	
			✓	1 (0)	7	3 (2)	✓	✓		
	✓	✓	✓	1 (0)	**8**	1 (0)	✓	✓		
✓	✓			2 (1)	9	1 (0)				✓
		✓	✓	3 (0)	10	4 (1)	✓	✓		
		✓	✓	3 (1)	11	2 (0)	✓			
	✓	✓	✓	3 (0)	12	6 (3)	✓	✓	✓	
		✓	✓	2 (0)	13	3 (1)	✓	✓		
			✓	1 (0)	**14**	1 (0)	✓			
		✓	✓	2 (0)	15	8 (6)	✓	✓	✓	
		✓	✓	5 (3)	**16**	2 (0)	✓			
		✓	✓	4 (0)	17	6 (2)	✓	✓		
		✓	✓	3 (1)	**18**	4 (2)	✓	✓	✓	
		✓	✓	2 (0)	**19**	4 (2)	✓	✓		
			✓	2 (1)	20	2 (1)	✓			
			✓	1 (0)	**21**	3 (2)	✓			
			✓	1 (0)	22	3 (2)	✓			
		✓	✓	2 (0)	**23**	3 (1)	✓	✓		
			✓	3 (0)	**24**	5 (2)	✓			
			✓	3 (1)	25	2 (2)	✓			
		✓	✓	4 (1)	26	5 (2)	✓	✓		
		✓	✓	4 (1)	**27**	4 (1)	✓	✓		
		✓	✓	5 (3)	**28**	3 (1)	✓			
			✓	2 (0)	29	3 (1)	✓			

## Data Availability

The data presented in this study are available on request from the corresponding author.

## Supplementary Materials

The following are available online at https://www.mdpi.com/2075-4450/12/2/148/s1, Figure S1: Larval length in mm for insect samples after different cooling periods, Figure S2: Larval length in mm for insect samples from the scene and the autopsy, Figure S3: Comparison of scene-based and autopsy-based PMI_min_ with temperatures of 20 °C and 25 °C. Table S1: Insect species found on the seven bodies used for the temperature profiles.
