# Supplementary material for: To Be There or Not to Be There, That Is the Question—On the Problem of Delayed Sampling of Entomological Evidence
Source: Insects. 2021 Feb 9;12(2):148. doi: 10.3390/insects12020148 (PMC7915408; doi:10.3390/insects12020148)
Supplement: Supplementary file 1 [file insects-12-00148-s001.pdf]

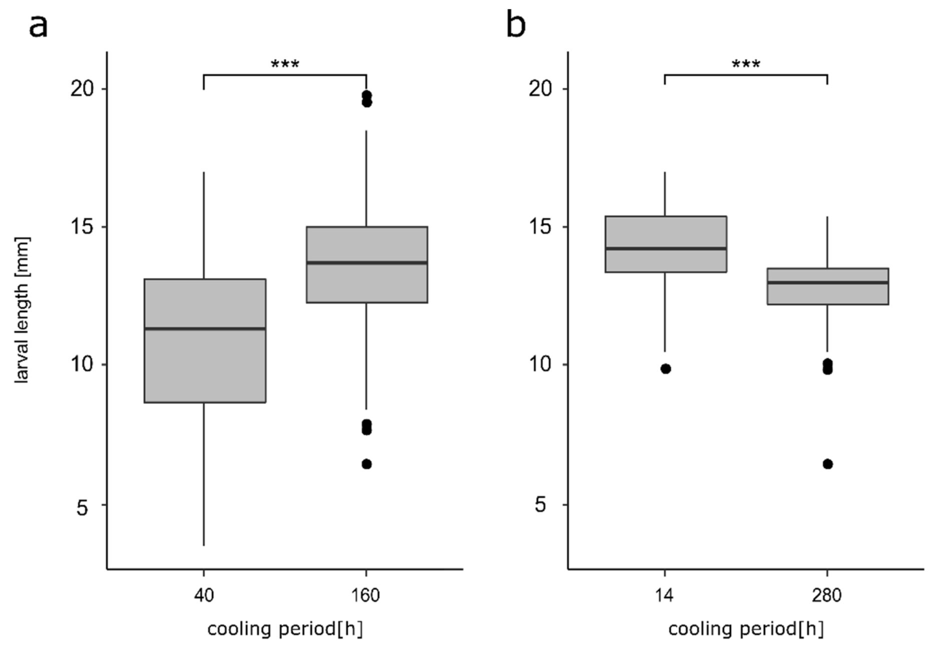

Figure S1: Larval length in mm for insect samples after different cooling periods. a) larvae of corpse 3 after 40 hours and 160 hours of cooling. b) larvae of corpse 4 after 14 and 280 hours of cooling. The asterisk indicates significant differences with $p < 0.001$.

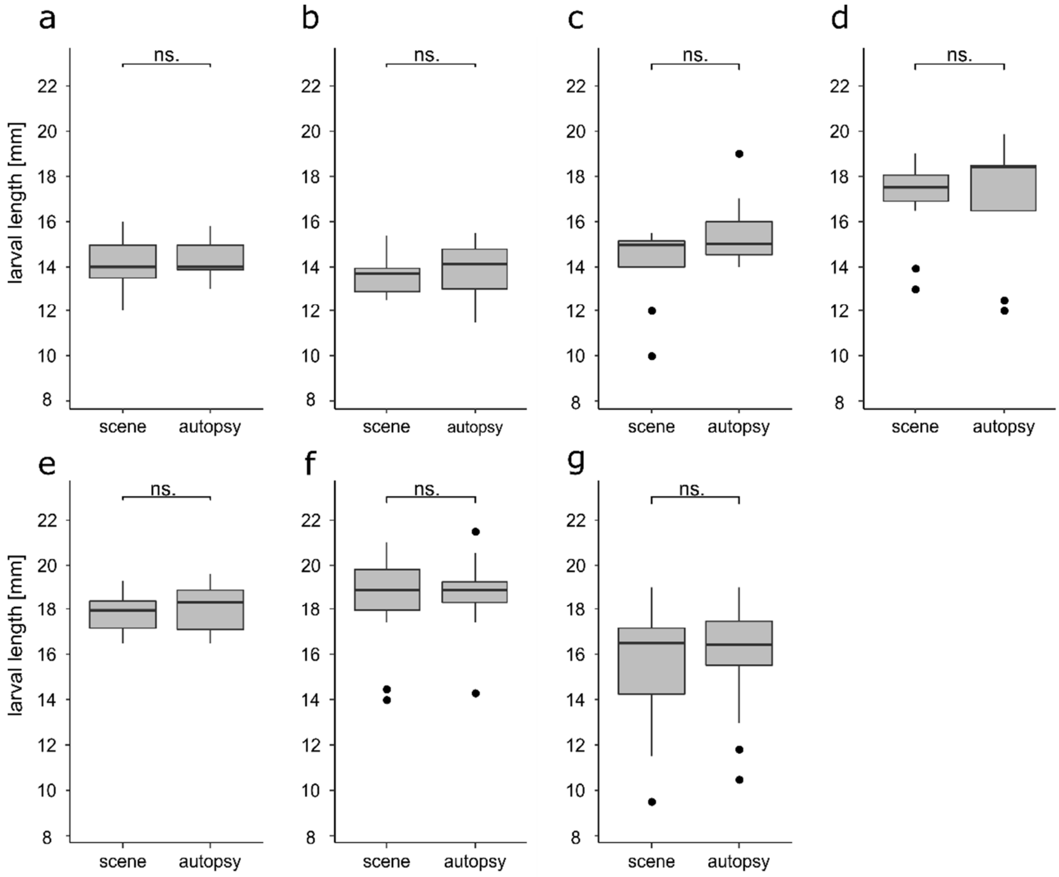

Figure S2: Larval length in mm for insect samples from the crime/death scene and the autopsy. **a)** larvae of *L. sericata* (both n = 16) from case 16 after 0 and 6 days of cooling; **b)** larvae of *L. sericata* (both n = 20) from case 18 after 0 and 7 days of cooling; **c)** larvae of *L. sericata* (both n = 16) from case 19 after 0 and 6 days of cooling; **d)** larvae of *C. vicina* (both n = 20) from case 8 after 0 and 10 days of cooling; **e)** larvae of *C. vicina* (both n = 10) from case 14 after 0 and 6 days of cooling; **f)** larvae of *C. vicina* (both n = 36) from case 28 after 0 and 7 days of cooling; g) larvae of *Ch. albiceps* (both n = 50) from case 21after 0 and 4 days of cooling

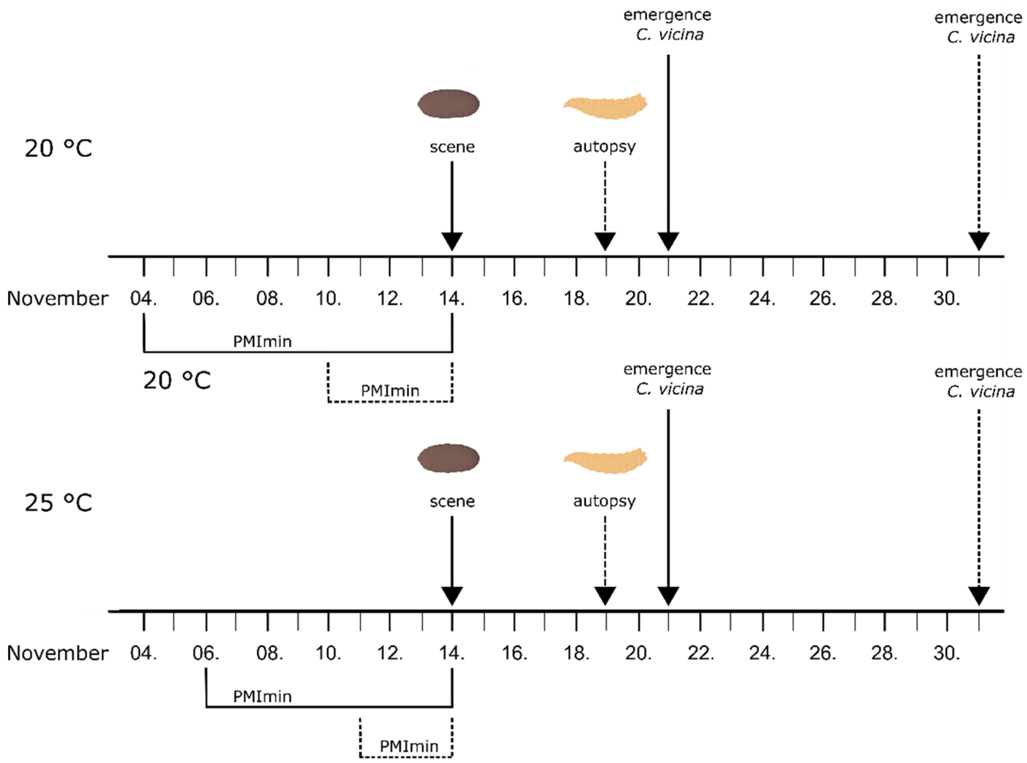

Figure S3: Comparison of scene-based and autopsy-based PMI_{min} estimations with temperature of 20 °C ad 25 °C. The time line shows the events (scene collection, autopsy collection) and the emergence of scene-collected and autopsy collected *C. vicina.* The solid line represent the scene-based PMI_{min} and the dashed line the autopsy-based PMI_{min}.

Table S1: Insect species found on the seven bodies. A checkmark indicates if the species was found on the body

**Species**	**2**	**3**	**4**	**5**	**6**	**7**	**8**
C. vicina	✓	✓					
C. vomitoria		✓					
Ch. albiceps			✓				
L. ampullacea	✓						
L. sericata	✓	✓	✓	✓	✓	✓	✓
P. regina		✓	✓	✓			✓
P. terraenovae		✓		✓			
S. argyrostoma		✓	✓				
